# Differing Approaches to Pain Management for Intrauterine Device Insertion and Maintenance: A Scoping Review

**DOI:** 10.7759/cureus.55785

**Published:** 2024-03-08

**Authors:** Mayisah Rahman, Connor King, Rosie Saikaly, Maria Sosa, Kristel Sibaja, Brandon Tran, Simon Tran, Pamella Morello, Se Yeon Seo, Yi Yeon Seo, Robin J Jacobs

**Affiliations:** 1 Medicine, Dr. Kiran C. Patel College of Osteopathic Medicine, Nova Southeastern University, Fort Lauderdale, USA

**Keywords:** intrauterine devices (iud), non-pharmacological treatments, pharmacological treatment, treatment guidelines, women's reproduction

## Abstract

Intrauterine devices (IUDs) are considered a reliable contraceptive option for women, but they can come with side effects. There is a disconnect in standard guidelines for IUD insertion within and without the U.S. The objective of this review was to address a gap in the literature regarding official procedures for pain management during IUD implantation. This scoping review was initiated using keywords to extract relevant articles from multiple databases: U.S. National Library of Medicine National Institutes of Health (PubMed), MEDLINE (Ovid), and Excerpta Medica dataBASE (EMBASE, Ovid). Initially, 457 articles were identified and after a rigorous screening and selection process, 37 articles were chosen to be further assessed to ascertain if they met the study’s inclusion criteria. Those 37 articles were further evaluated fully to check for relevancy. From that process, 19 articles were chosen for the review, and all passed quality assessment evaluations using the JB Appraisal Tools. To best address the research question, the data from the 19 articles were divided into three categories: 1) circumstantial factors, 2) non-pharmacological methods, and 3) pharmacological methods. Circumstantially, women with previous vaginal deliveries experienced the lowest pain during the procedure, and nulligravid (never pregnant) women experienced the most pain. Lower pain scores were reported by lactating women compared to non-lactating. Black women experienced the most anticipated pain compared to other races. Regarding non-pharmacological methods, different insertion techniques, tools, and the use of a cold compress were found to not affect the level of pain during IUD insertion. Lastly, it was shown that pharmacological methods such as lidocaine gel, lidocaine paracervical block, and lidocaine combined with either diclofenac or prilocaine decreased pain scores at different time stamps of the procedure. Also, oral ketorolac and a vaginal combination of misoprostol and dinoprostone helped reduce pain. Findings from this scoping review revealed a lack of uniformity across practices when performing IUD insertions, possibly due to differences in procedures across circumstantial factors, non-pharmacological methods, and pharmacological methods. More research is needed to investigate the intricacies of pain with IUD insertion. Moving forward, especially following a potential increase in the use of IUDs after the reversal of Roe v. Wade, establishing this gap may lead to a more refined standardized protocol to mitigate pain with IUD insertions.

## Introduction and background

In June 2022, the United States (U.S.) Supreme Court overturned the ruling of Roe v Wade, a monumental case permitting women of reproductive age, the right to have an abortion. While long-term implications are being explored, the decision will potentially have an impact on attitudes and practices toward contraception. For instance, an analysis of searches post-verdict depicts an increased interest in intrauterine devices (IUDs) among Google users [1]. Regardless of this decision, IUDs have grown in popularity in recent years, becoming the fourth most favored method of birth control [2]. Serving as a long-acting reversible contraceptive, increased desire results from its effortless management after placement and effective fertility after removal [3]. However, when discussing the negative aspects of IUDs that might discourage women from having one inserted, the major reason for not wanting one was the fear of pain [4].

It has been reported that some women experience anxiety about the severity of pain and discomfort that can be associated with IUD insertion [5]. Even providers inserting the device underestimate levels of pain by half when compared to women going through the procedure, which could explain the lack of universal technique and counseling [6]. When observing patients, nulliparous women experienced more pain compared to patients who had gone through vaginal delivery, indicating a group to target for pain control [7,8]. Considering pain management during the IUD placement, inserting a hysterometer into the cervix proved to be the most uncomfortable step, providing an avenue for stage-specific pain prevention measures [9]. If this barrier regarding the fear of pain is overcome, continuous pain still proves to be a reason for IUD removal [10]. Given the possibility of the increased use of IUDs, it becomes beneficial to look at the perception of pain with the procedure and existing protocols to combat this barrier.

Concerns with discomfort have led to various methods attempting to reduce it before, during, and after IUD insertion. Despite ongoing research, different approaches demonstrate a lack of effectiveness in pain prevention. To illustrate, a cold compress on the abdomen, although attainable, did not show improvement in distress during the procedure [11]. Prophylactically, non-steroidal anti-inflammatory drugs (NSAIDs) such as 800mg of ibuprofen, compared to a placebo, had a similar effect in not reducing pain [8]. NSAIDs have also been explored in post-insertion care; however, they did not deliver promising results to improve discomfort and may even induce side effects with medications such as misoprostol [5,12-14]. Drug combinations including naproxen and lidocaine were meant to help during and after the visit yet did not show any difference in satisfaction compared to other methods [15].

Although the previous measures have been ineffective, varying alterations and combinations have presented as practical. For instance, certain formulations of lidocaine and naproxen can have effects on specific groups; however, tramadol demonstrated a greater impact [16]. A mixture of local anesthetics involving lidocaine and prilocaine was used to form a topical analgesic cream. This cream reportedly aids in the most painful part of the procedure: hysterometer insertion, IUD insertion, and tenaculum use [9,17]. Similarly, 10% lidocaine spray improved discomfort during insertion in a non-invasive approach [18]. Another preparation of lidocaine in the form of a paracervical block was useful in pain reduction during and five minutes after placement it was also accompanied by pain during block administration [19]. While intramuscular ketorolac did not reduce pain during insertion, it decreased pain scores 15 minutes following it [20]. The aforementioned analgesics provide an outlook into the ongoing exploration of IUD pain management.

Considering the current event in reproductive legislation, the potential increase in contraceptive use highlights IUDs as a reproductive choice for women [1]. Despite its benefits, an obstacle to IUDs is the potential pain associated with insertion and use [4]. While there have been studies on pain management measures, many yielded clinically insignificant results [8]. As for the medications described as functional, many come with adverse effects or are dependent on different formulations that have not been universally specified. Overall, this review summarizes individual methods and addresses the gap in consistent and practical protocols to be used by all providers. This study aimed to review research related to IUD pain and subsequent approaches for pain management to address the question: What is the current pain management protocol for IUD implementation and maintenance in the U.S.?

## Review

Methods

This scoping review was organized to answer the question: What are the current pain management protocols for IUD implementation and maintenance? Additional goals of this review are to 1) clarify the timing within pain management protocols prior to, during, and after implantation and 2) describe patient and physician attitudes about pain with IUD insertion.

Inclusion/Exclusion Criteria

Articles included in this review were searched using the PCC (population, concept, and concept) framework and were screened for including studies that 1) included women of childbearing age, 2) described pain management protocols to ameliorate pain with IUD insertion and, 3) had findings relevant in a post-Roe v. Wade era. Included studies had to be peer-reviewed, published between January 2015 and September 2022, and discussed pain management protocols for IUD insertion. There are many outcomes to receiving an IUD that, while important, are not related to pain during the IUD insertion (e.g., actinomycosis, IUD migration) and were thus excluded. Only randomized controlled trials, cohort studies, and case studies were included as these studies contain a detailed pain management protocol that could be theoretically implemented in a clinical setting. Studies were limited to those that included women who were 18-65 years old or of childbearing age who did not have the following: 1) any other comorbidities or medical conditions (PID, adenomyosis, etc.) and 2) any other concurrent contraceptive use. This specific population was targeted to focus on pain that is derived solely from the IUD insertion procedure with minimal confounding factors. Additionally, the study population used IUDs as their primary contraception choice pre- and post-Roe v. Wade. 

Search protocol

Since pain management with IUD insertion is a fairly nascent topic, it was necessary to maximize search results while maintaining a narrow scope. To accomplish this, all synonyms and acronyms of IUD were included along with the non-specific word “pain.” To filter for appropriate studies, these terms were used for titles and abstracts only. This generated a more nuanced Boolean operator phrase: (Intrauterine device OR IUD OR IUC OR intrauterine device insertion) [Title/Abstract] AND (pain)[Title/Abstract] AND ((ffrft[Filter]) AND (fft[Filter]) AND (2015/1/1:2022/8/4[pdat])). This phrase was used to search and compile relevant articles from the U.S. National Library of Medicine National Institutes of Health (PubMed), MEDLINE (Ovid), and Excerpta Medica dataBASE (EMBASE, Ovid) databases. These databases were selected to maximize the number of controlled trials and case studies while including articles outside of the search criteria that could prove useful. The Digital Object Identifier (DOIs) or PubMed identification numbers (PMIDs) of compiled articles were saved as an Excel (Microsoft Office) file and uploaded for review to Rayyan software (Ouzzani et al.). All searches were performed in September 2022.

Screening and selection

**Figure 1:**
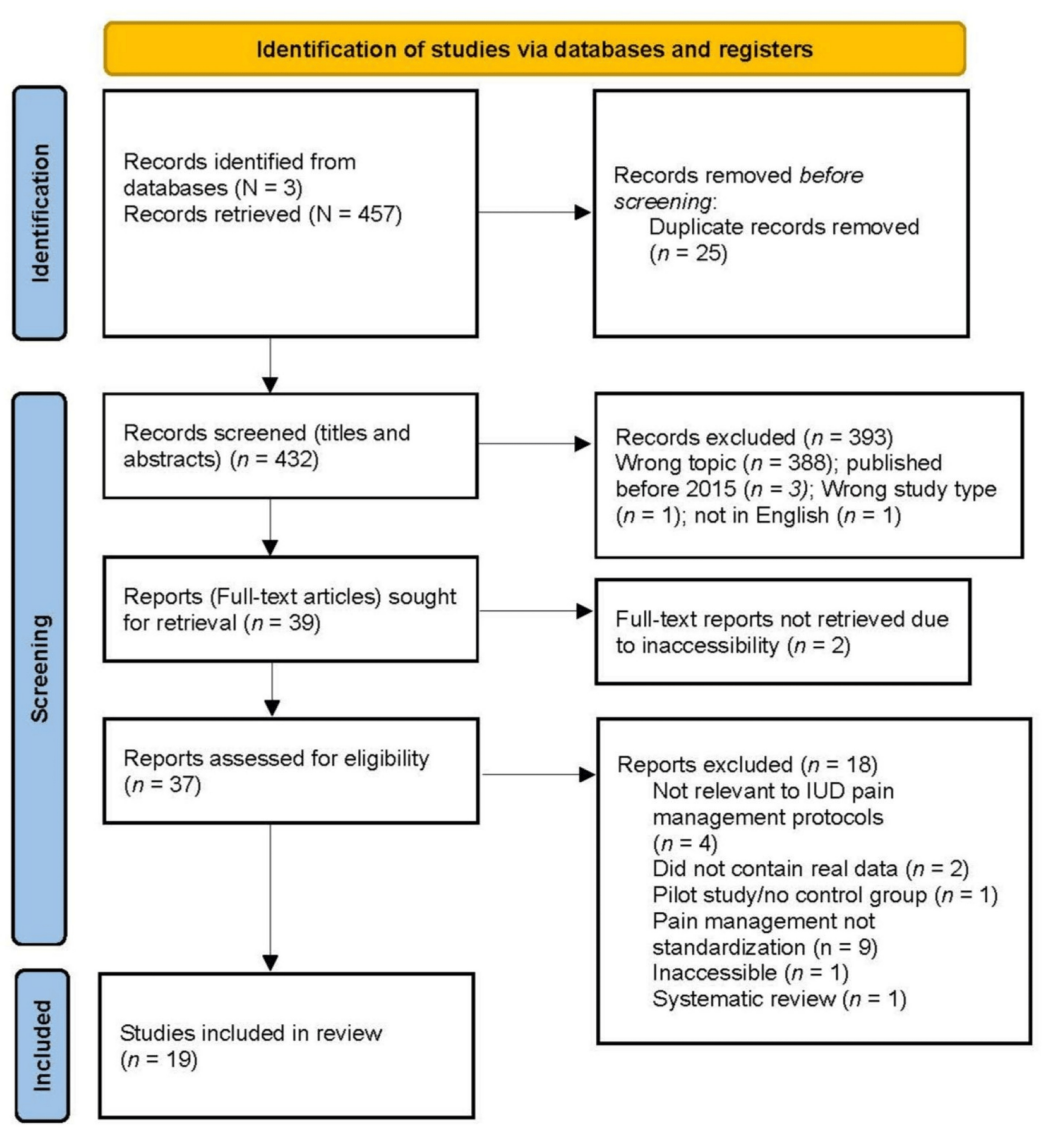
PRISMA flow diagram PRISMA: Preferred Reporting Items for Systematic Reviews and Meta-Analyses IUD: Intrauterine device

The initial search yielded 457 articles which were then uploaded to a software program that aids in the screening process for collaborative systemized reviews. After removing 25 duplicate articles, 432 articles remained for screening. All members of the research team reviewed the titles and abstracts to determine which articles met the study’s inclusion criteria and which were appropriate for full-text reviews. The team collaboratively discussed each selected article to rectify any selection conflicts between reviewers, resulting in 37 articles for final independent review by two members of the team. Articles resulting in conflict between the two reviewers were evaluated by a third reviewer. All three reviewers then discussed each article for which there was disagreement on inclusion and reconciled; 19 articles were ultimately retained to be included in the review (Figure 1).

Data extraction and synthesis

Data were extracted from the 19 articles according to a data-charting form that was jointly developed by the research team. Each reviewer independently extracted data from all fully reviewed articles, discussed the extracted data, and recorded any additional data that was potentially important for inclusion. The data-charting form was updated continuously in an iterative process to better match the evolution of the review’s topic. Any inconsistencies in extracted data were discussed between reviewers until conflicts were resolved and all extracted data was verified.

Extracted data included all aspects of the pharmacological (dosing, timing, method of application) and physical (technology or manipulation used) pain management protocols, respective outcomes of the protocols (values of improvement, lack of improvement), patient demographics (age, parity status), study location, and any assessment of patient and physician attitudes. All protocols that improved pain were organized into a table format detailing the exact methodology and population demographics. If applicable, any patient or physician attitudes on the pain management protocols were added to the table. 

Results

The initial literature search from the databases resulted in a total of 457 articles. Following the removal of duplicates and eligibility assessment, 19 studies were deemed appropriate for this review. Evaluation of the studies was categorized based on the impact of 1) circumstantial factors, 2) non-pharmacological methods, and 3) pharmacological methods on pain associated with IUD insertion.

Circumstantial factors

Of the included studies, four of them explored how different patient circumstances influenced pain scores regarding IUD insertions [7,21-23]. Among these studies, some explored the involvement of psychosocial factors in anticipated pain [7,21], while others examined the different experiences of nulligravidas and parous women during the procedure [22,23].

Gathering data on demographics, sexual/gynecologic history, and mood, the first study identified predictors of anticipated pain [21]. Multivariate analysis demonstrated that race was the most significant covariate in predicting anticipated pain. Within this covariate, univariate analysis showed that Black participants had a median anticipated pain score of 68 (IQR 52-83) compared to White participants who had a score of 51 (IQR 35-68), and participants of other races with a score of 64 (IQR 36-73), suggesting that Black race was the only factor found to be associated with higher levels of anticipated pain [21]. 

Another study found no correlations between personal circumstances, demographics, and actual or expected pain [7]. Findings suggested, rather, that there may be a link between pain and previous gynecologic history, such as vaginal delivery. The median actual pain experienced by women during insertion, with a score of 4, was significantly lower than the expected pain median, a score of 6 (p<0.001). Women who did not have a history of vaginal delivery had significantly higher actual pain at a median score of 6 (IQR 3.5-7.5) than women with a history of vaginal delivery at a median score of 3 (IQR 1-5) (P<0.001) [7].

Fouda and colleagues found that pretreatment impact was not clinically relevant [22]. Instead, the study showed that lower mean pain scores during IUD insertion were reported in women with previous vaginal deliveries, scoring 3.29±1.05, compared with women who delivered only by cesarean section scoring 4.41 ± 1.24 (p=.001) and lower scores in lactating women, with a score of 2.55 ± 0.96, compared with non-lactating women, scoring 4.04 ± 0.96 (p=.001). Women with IUD insertion within six months from their last vaginal delivery also showed a lower score of 3.02 ± 1.26 compared to women with IUD insertion more than six months from their last vaginal delivery with 3.96 ± 0.95 (p=.001) [22]. 

Similarly, the final study under circumstantial factors explored how gynecological history impacts pain scores [23]. Multiple linear regression analysis demonstrated that 29.5% of the variability in the pain score was explained by nulligravid or women who had elective cesareans and difficulty at IUD insertion. Nulligravid women presented a higher mean pain score of 6.6 ± 2.0 when compared to women with elective cesarean delivery, 5.5 ± 2.1, and women with previous vaginal delivery, 3.9 ± 2.4 (p < 0.001). These two groups with high pain scores (nulligravid and cesareans) were also more likely to have pain classified as moderate or severe (in relation to absent or mild) than women with previous vaginal delivery (p < 0.001) [23]. 

Non-pharmacological methods 

Four articles included various methods to prevent or reduce pain upon IUD insertion. They evaluated non-pharmacological interventions including different placement methods, instruments, and therapies to alleviate pre and post-insertional discomfort [11,24-26].

Lambert and colleagues aimed to determine how different methods of placement, such as “slow” placement and the “cough” method, affected the pain score for patients [24]. Slow placement showed a median pain score of 44 (IQR= 21, 63) while cough placement had a mean score of 32 (IQR 19, 54) for tenaculum placement (primary outcome). Univariate analysis of VAS values (0-100mm) showed that the scores did not differ by group, with a median pain score for overall pain of 62 (IQR=48, 84) in the slow placement group and 54 (IQR=32, 71) in the cough method group (p=0.12). Provider satisfaction (secondary outcome) was not associated with one method of placement (p=1) [24]. In consideration of the primary and secondary outcomes, neither method proved to be superior. 

Rather than placement, another study compared methods of insertion in terms of pain experienced and satisfaction [25]. The methods involved were the single-toothed tenaculum and a Littlewoods forceps to stabilize the cervix for IUC fitting. Univariate analysis of VAS values (0-100mm) showed less pain with insertion using the direct method (mean=19.9 mm; SD=22.5) compared to the standard method (mean=33.4 mm; SD=29.3). A multivariate analysis of insertion pain predictors showed that using the direct method was associated with a lower VAS value for insertion pain (8.3 mm 95% confidence interval -14.3, -2.3) [25]. The analyses determined that the direct method was significantly less painful than the standard and resulted in fewer adverse effects. 

One study assessed the pain and satisfaction of IUD insertion when using either the single-toothed tenaculum or the Littlewoods forceps [26]. Mean VAS pain scores were similar between the two groups at forceps application, at IUC insertion, and at 5 minutes post-IUC insertion. Mean VAS scores were similar at forceps application (p=0.52), IUC fitting (p=0.10), and at 5 minutes (p=0.32). The tenaculum group saw a difference in pain scores at 10 minutes (p=0.01). Satisfaction from physicians was similar for both tools and bleeding was similar as well (p=0.49) [26]. After adjusting for multiple tests, the differences between the tools were not clinically relevant. 

Whereas the other studies in this category explored procedural adjustments, Hylton et al. examined the efficacy of a non-pharmacological therapy during IUD insertion, known as the cold compress [11]. Participants receiving the cold compress rated the insertional pain as a 4.3 while the control group participants rated it at 4.6 (p=0.805). Regarding post-insertional pain, participants receiving the cold compress scored a 3.4 compared to the 3.5 in the control group, demonstrating the cold compress’ inability to reduce pain.

Pharmacological methods

Of the articles in this review, 12 evaluated pharmacological methods to reduce pain with IUD insertion. These methods included local anesthetics (lidocaine), NSAIDs, and prostaglandin analogs, all formulated and administered in different ways. For instance, lidocaine was formulated as a gel, injection, spray, and in-solution and administered as a paracervical block or directly onto the external cervical os. Table 1 reports the data on the pharmacological methods used.

**Table 1:** Pharmacological methods to decrease pain with intrauterine device (IUD) insertion. *Administered with another drug **Did not decrease pain with intrauterine device (IUD) insertion, but reduced pain at another measured time point (i.e., cervical traction)

Drug	Method	Pain decrease with IUD insertion
Lidocaine	Cervical gel and spray	No**
	Intrauterine solution ± oral naproxen*	No
	Cervical spray	Yes
	Cervical gel (+oral diclofenac)*	Yes
	Cervical cream (+prilocaine)*	Yes
Lidocaine	Paracervical block	Yes
	Paracervical block	Yes
	Paracervical block	No
Non-steroidal anti-inflammatory drugs (NSAIDs)	Oral Naproxen ± intrauterine lidocaine*	No
	IM ketorolac	No**
	Oral ibuprofen	No
	Oral ketorolac	Yes
Prostaglandin analogs	Vaginal misoprostol and dinoprostone	Yes
	Oral misoprostol	No

Seven out of 12 studies resulted in a significant decrease in pain on VAS values during IUD insertion: 10% lidocaine cervical spray [18], combination of two 50 mg diclofenac potassium oral tablets and 2% lidocaine cervical gel [22], a 5% cream mixture of 25 mg lidocaine and 25 mg prilocaine per gram of cream applied to the cervix [9], 20 mL 1% lidocaine paracervical block [19], 6 mL 2% lidocaine paracervical block [27], 20 mg ketorolac oral tablets [28], and vaginal tablets of 200 μg misoprostol and 3 mg dinoprostone [29]. The successful trials primarily involved applying medication directly to the cervix or using the medication within the vagina rather than systemically. Two trials failed to demonstrate significant pain reduction during IUD insertion, but pain was reduced during other measured time points, such as during cervical traction [30] and 5 and 15 minutes following IUD insertion [20]. Strangely, these drugs, lidocaine as a cervical gel and spray [30] and intramuscular ketorolac 30 mg [20], had reduced pain in other studies or other formulations. Additionally, not all trials that utilized paracervical blocks with lidocaine significantly reduced pain with IUD insertion [19,27,31]. Table 2 reports the characteristics of the 18 articles included in this review.

**Table 2:** Summary of the 19 articles included in the review. N: Sample size; IUD: intrauterine device; EMLA: eutectic mixture of local anesthetics; cc: cubic centimeter

Authors	Study Design	Sample	Study Aim	Specialty	Findings	Recommendations	Limitations
Brima (2015) [7]	Analytical Cross-sectional Study	N = 89	Compare the expected and actual pain experienced with the placement of IUD, and to evaluate if these are associated with personal circumstances or impacted their satisfaction with the procedure.	Camberwell Sexual Health Clinic based at King’s College Hospital, London	Expectation of pain prior to the IUD insertion was high for all women, but for those who have had vaginal deliveries their expected pain was significantly higher than actual pain. When comparing women who had not had a previous vaginal delivery with those who have, actual pain experienced was much higher.	Assessing the participants anxiety before the placement of the IUD. Additional research on types of pain management strategies for IUD placements.	The use of a more objective numerical scale versus a visual analog scale (VAS) or a tool that requires administration by the researcher to the patient. The small convenience sample.
Bednarek (2015) [8]	Randomized Controlled Trial	N = 202	Evaluate the effectiveness of 800mg ibuprofen in reducing pain with intrauterine device (IUD) insertion among US women.	Department of Obstetrics and Gynecology, Oregon Health and Science University, Portland, OR	Mean and median pain scores did not differ between placebo and ibuprofen when nulliparous and parous women were analyzed independently.	Concentrate on alternate strategies to reduce pain and improve satisfaction with IUD insertion among nulliparous women. Assess pain and drug absorption at different time points.	The study failed to enroll the number of participants it had originally estimated. It also did not address pain in the hours after placement. Only one time point was evaluated for drug absorption.
Tavakolian (2015) [9]	Triple Blind Clinical Trial	N = 92	Evaluate the efficacy of a eutectic mixture of local anesthetics (EMLA; that contain 25mg lidocaine and 25mg prilocaine) at reducing pain during IUD insertion at multiple points during the procedure	Department of Midwifery, Shahid Beheshti University of Medical Sciences, Tehran, IR Iran	Use of EMLA cream significantly reduced pain at multiple points during the IUD insertion procedure. Patients in both the experiment and control group found hysterometer insertion as the most painful part of the procedure.	Expand the use of EMLA cream during IUD insertion procedures	Demographic data was presented as mean values instead of the raw number of participants that fit into each category. This obfuscates exactly who can benefit from using this method. Unclear if one or more clinicians performed the IUD insertion procedure.
Hylton (2020) [11]	Randomized Controlled Trial	N = 69	Determine whether use of cold compress on abdomen during IUD insertion reduces pain	University gynecology clinic Adults	No reduction in IUD insertional pain with cold compress	Consider perceptions of pain and in those with pain before procedure Assess role of both tenaculum placement and diameter of device placed in procedure-related pain as well as approaches to transpose anxiety-related to IUD insertion	Lack of documented tenaculum placement Lack of confirmation of timing of cold compress application Did not stratify BMI (which may have affected impact of cold compress) Did not stratify for parity or delivery type
Miles (2019) [15]	Randomized Controlled Trial	N = 160	Evaluate oral naproxen and intrauterine instillation of lidocaine for analgesia with intrauterine device (IUD) as compared to placebo	Walter Reed National Military Medical Center outpatient obstetrics and gynecology clinics	Naproxen with or without intrauterine lidocaine does not reduce pain with IUD placement	Include separate arm with no intervention (instillation of saline, usage of 18-gauge Angio catheter) other than IUD insertion, limit subjects to nulliparous women	Possible alternative treatment modalities other than IUD insertion, study not randomized to type of IUD
Aksoy (2016) [18]	Randomized Controlled Trial	N = 200	Study the effectiveness of 10% lidocaine spray to the cervix in reducing pain during IUD insertion.	Department of Obstetrics and Gynecology, Kayseri Education and Research Hospital, Kayseri 38010, Turkey	Lidocaine 10% spray is shown to be effective in lowering pain levels during IUD insertion when compared to placebo.	Perform a larger scale study involving other types of topical anesthetics in order to assess lidocaine effectiveness and optimal dosage.	Lack of a non-treatment group in addition to the treatment and placebo groups. The study also did not assess pain levels after the IUD is inserted. Finally, the study was only conducted in parous women, excluding nulliparous women.
Mody (2018) [19]	Randomized Controlled Trial	N = 64	Determine if a 20-cc buffered 1% lidocaine paracervical block could reduce pain during IUD placement	University of California, San Diego and Planned Parenthood of the Pacific Southwest	The 20-cc buffered 1% lidocaine paracervical block was able to decrease pain during the IUD placement, with uterine sounding, and 5 minutes after the placement. The overall perception of pain was lower when a block was administered compared to when no block was given at all.	Expanding the generalizability of the population by increasing the diversity in the age, education level and race of the participants to represent the national population.	The study had a lack of diversity when it came to education, age and race of the participants. In addition, not all types of IUDs where included, such as the LNG-52 mg IUD (Liletta®).
Ngo (2015) [20]	Randomized Controlled Trial	N = 67	Evaluate intramuscular ketorolac compared to placebo saline injection for pain control with IUD placement	University of California San Diego Women's Health Clinics	Ketorolac does not reduce pain with IUD placement but does reduce pain at 5 and 15 minutes after placement	Additional studies on efficacy of naproxen during and after IUD placement, larger sample size	Study was not powered to detect pain score differences less than 2.0cm or subgroup analysis by parity or other subgroups like IUD type, small sample size, participants were not followed past 15 minutes of injection to minimize clinic flow interruptions, unblinded staff administering study forms (could have caused bias), ketorolac may not be available in all clinics, painful intramuscular injection
Hunter (2020) [21]	Randomized Controlled Trial	N = 93	Identify factors that are associated with anticipated pain with IUD insertion in young women and adolescents	Three academic family planning clinics	Black race was only factor found to be associated with higher levels of anticipated pain	Recommend providers discuss with all women their anticipated pain, anxiety, pain management preference, and actual pain when undergoing IUD placement. Additional training in racial disparities and racial sensitivity.	Difference in anticipated pain between minority populations can be complex and multifactorial. Study population was only young adults therefore may not be generalizable to older populations. Secondary analysis which limited analysis to previously recorded data.
Fouda (2016) [22]	Randomized Controlled Trial	N = 90	Determine if diclofenac in combination with 2% lidocaine gel can reduce pain scores during the IUD insertion procedure.	Department of Obstetrics and Gynecology at Cairo University, Egypt.	Participants receiving diclofenac and lidocaine gel combo had lower pain scores during tenaculum application and IUD insertion. Pain scores were lower in women with previous vaginal deliveries compared to those who got a C-section.	Conduct additional research with both nulliparous and multiparous women as participants and have 1 or 2 gynecologists perform the insertions. Consider the diclofenac and lidocaine combo as a step in the pain management protocol.	None of the participants were nulliparous. 8 gynecologists completed the procedures, so it could have resulted in different experiences with different gynecologists. Only one type of IUD was used in the study, so the results may be different with other IUDs.
Chaves (2021) [23]	Prospective Single Cohort Study	N = 413	Compare pain scores during the insertion of a levonorgestrel releasing intrauterine system in women who have never given birth, women with previous vaginal delivery and women with previous cesarian delivery	Family Planning Service, Department of Obstetrics and Gynecology, Hospital das Clinicas of the Federal University of Minas Gerais (UFMG), Belo Horizonte, MG, Brazil	Nulligravidas women had the highest mean pain scores among the three groups and nulligravidas women and women who had undergone elective cesarian delivery mostly classified their pain as moderate or severe.	Further investigating the differences in pain response among nulligravidas women, women with only vaginal deliveries and women with only cesarian deliveries and evaluating the impact that pain management can have among the groups	Risk of informational bias due to both gynecologist and resident physicians collecting data. The clinician performing the procedure was not blinded to what group a participant belonged to, creating possibility of bias. The study also did not take into consideration the role of past experiences such as obstetric history and sexual abuse.
Lambert (2020) [24]	Randomized Controlled Trial	N = 66	Compare pain reduction between "slow" and "cough" techniques for tenaculum placement	University gynecology clinic Adults	Neither method is superior for pain reduction or provider satisfaction	Larger study to demonstrate clinically significant differences in experience of pain	Unmeasured confounders such as anxiety level and use of anxiolytics
Bastin (2019) [25]	Prospective Observational Study	N=281 direct method; N =254 standard method	Compare the pain experienced using the direct method vs the standard method of IUD placement	General practitioner, gynecologist, and midwives	Less pain with insertion using the direct method of insertion vs the stand method of insertion. Additionally, using the direct method led to fewer adverse effects up to 6 months post insertion	Further studies to examine the contraceptive efficacy of IUDs placed with the direct method.	Study was observational and not a randomly controlled trial. Direct method group tended to utilize more drug-free strategies introducing a possible confounding variable.
Speedie (2016) [26]	Randomized Controlled Trial	N =100	Compare the single-toothed tenaculum to the Littlewoods forceps in regard to pain and ease of use during the IUD insertion procedure.	Community Sexual and Reproductive Healthcare Clinic in the United Kingdom.	No difference in pain scores during insertion and 5 minutes post-insertion. There was a difference 10- minute post-insertion; however, the scores were very low by 10 minutes with either forceps. No difference in the ease of use, with both being easy to use.	Either one of the forceps is an effective and appropriate tool for the insertion procedure, so the type of forceps might not be considered a factor when creating a pain management protocol. A new study with a larger sample size should be considered.	The study had a low sample size of only 100 participants. Some participants (30%-36% of participants in each arm) had already taken oral analgesia prior to the procedure (this was corrected for but did not mention how).
de Oliveira (2021) [27]	Randomized Controlled Trial	N = 100	Compare the effectiveness of 550 mg of naproxen sodium and lidocaine 2% intracervical block in lowering pain during IUD insertion.	Family Planning Service, Department of Obstetrics and Gynecology, Hospital das Clínicas of Federal University of Minas Gerais (UFMG), Belo Horizonte, MG, Brazil	Lidocaine 2% intracervical block was found to be significantly more effective than 550mg of naproxen sodium in lowering pain during IUD insertion in young women.	double-blind study and match physician’s skill levels.	The medication option used was not blinded to either the physicians performing the procedure or the patients. In addition, physicians' experience levels were not the same, meaning most of them were residents, which could interfere with the pain levels.
Crawford (2017) [28]	Randomized Controlled Trial	N =72	Evaluate if oral ketorolac provides effective pain relief during placement of an IUD for contraception.	OhioHealth Riverside Methodist Hospital, Columbus, OH, USA	Oral ketorolac given 40 to 60 minutes prior to IUD insertion is effective in reducing pain during IUD deployment, overall pain, and pain 10 minutes after IUD placement.	Evaluate pain at different points of the procedure. Collect adverse effect data or post procedure pain medication use. Increase generalizability by utilizing more sites.	Lacked observation 30 and 60 minutes after procedure to consider the peak analgesic effects. LNG-releasing IUD was not available at the participating offices at the time of the study and thus application to this device may not be consistent with the devices studied. As this trial was completed at one site, this limits generalizability
Ashour (2020) [29]	Randomized Controlled Trial	N =129, N=43 Misoprostol, N = 43 dinoprostone	Compare the effect of Misoprostol vs dinoprostone vs placebo administration with IUD placement and its relative pain scale and ease of insertion	Family planning clinic of a tertiary referral hospital Cairo, Egypt	There was less pain and increase of ease in women who was administered Misoprostol and dinoprostone versus those who received the placebo	Further studies are needed to look at the side effects of these medications as prostaglandin analogs	This study only accounted for 2 prostaglandin analog and one type of IUD. (Copper T380A)
Torky (2017) [30]	Quasi-Experimental ( Prospective Multicentre Non-Randomised Comparative Study)	N = 420, N= 140 Lidocaine Gel, N= 140 Lidocaine Spray, N = 140 Placebo	Compare the effects of lidocaine gel vs spray on the perceived pain of IUS insertion	Air force Specialized hospital (New Cairo, Egypt), University Hospital (Giza Egypt), Al-Galaa Teaching hospital (Cairo, Egypt)	There was no significance with the use of lidocaine spray nor gel in comparison to the placebo in regard to IUD insertion. But there was significance when it came to pain during cervical traction.	Timing and dose should be considered for the application of analgesic for the optimal pain relief for IUD insertion.	This study was conducted in three different hospitals, participant number was not noted from how many participated from each respective hospital.
Elkhouly (2017) [31]	Randomized Controlled Trial	N = 200	Compare the use of Lidocaine, Misoprostol, and an NSAID to reduce pain during IUD insertion and the advantages of using one drug over another	Outpatient clinic of Menoufia University Department of Obstetrics and Gynecology	Similar mean pain scores during the IUD insertion procedure and 15 mins after in the groups who were administered an analgesic and the placebo group.	Additional research on perceived vs actual pain during IUD insertion, the effect of pain management when the insertion is performed by an inexperienced clinician and the use of pain management drugs when the procedure is performed without the use of a tenaculum	Researchers did not take into consideration whether the insertion was done in a routine or emergency setting, the position of the uterus in each participant, the experience level of the practitioner performing the procedure, and perceived pain of each participant. The clinicians performing the procedure were also not blinded creating the risk of bias when conducting the insertion

Discussion

While currently there are limited standardized guidelines in the U.S. for managing pain during or following IUD insertion, investigations to evaluate pain management protocols for IUD insertion are increasing. The results of this scoping review suggest potential avenues within the categories pharmacologic, non-pharmacologic, and circumstantial factors for future investigation. Results of this review suggest that a multifaceted approach using the cough method of placement [24], combined with the direct method [25] of IUD insertion and accompanying it with a local anesthetic or prostaglandin analog might be optimal. These pharmacological methods would be used directly within the vagina [29] or applied directly to the cervix [9,18,22] and could be accompanied by an oral NSAID as needed.

Circumstantial factors

Nulliparity has been demonstrated as a clinical determinant in pain perception during IUD insertion. Parous women have consistently reported diminished pain levels in comparison to their nulliparous counterparts and women who have not undergone vaginal deliveries, irrespective of the employed treatment modalities. Notably, heightened anticipatory anxiety preceding the procedure has been correlated with elevated pain scores, as indicated in the study conducted by Brima [7].

Race emerged as a significant factor influencing pain anticipation and reported pain levels during the procedure, with Black women reporting higher levels of both in contrast to their White counterparts within a shared demographic context [21]. The unexplored psychosocial and ethnic variations underscore the importance of incorporating these factors into future research endeavors. For instance, further exploration regarding effective pain control for various demographic populations including race and age can reveal the obstacles preventing a universal pain management protocol and narrow down various options. Further research on psychological factors, especially anxiety and its impact on pain scores, can help reveal accessible improvements not just pharmaceutically, but in the physical environment, demeanor, and resources of clinics. Such considerations are paramount for the development of more nuanced and individualized protocols, catering to specific patient populations, thereby facilitating the establishment of optimal clinical practices.

Non-pharmacological methods

No statistical difference in the degree of pain was found in using different stabilizing forceps during the procedure, Littlewoods or single-toothed tenaculum forceps [26]. Additional therapies can also be investigated to alleviate insertional discomfort, including cervical priming through medications like prostaglandins, catheters, or even home remedies and supplements. Reported clinical trials often investigated these drugs and methods in isolation, emphasizing the importance of future studies to include a multifaceted approach to establish best practices.

Pharmacological methods

The studies reporting lidocaine administration via paracervical block appear to be more effective compared to pharmaceutical counterparts considered in other studies such as intramuscular naproxen [20,27], diclofenac alone [22], ketorolac [20], and prophylactic oral ibuprofen [8] when looking at significant pain decrease with IUD insertion. In addition, other studies that combined lidocaine with other pharmaceuticals have shown positive results, such as 50/50 Lidocaine and Prilocaine 5% cream [9], and 2% Lidocaine gel with 30 mg Diclofenac potassium [22]. Administration of oral prostaglandins (misoprostol and dinoprostol) promoting cervical relaxation has also resulted in favorable responses to IUD insertion [29]. Systemic methods of analgesia did not produce effective pain relief, meaning it is important to have the drug act directly in the vagina and on the cervix to manage pain during IUD insertion. Although systemic methods may have lacked results during insertion, future research can further explore the timeline of pain from the anticipatory stage to the subsequent stages to assess the value of these methods.

Limitations

This review has several limitations. Some of the studies included in the review had small sample sizes, thereby increasing the risk of error. The search criteria also failed to include a specific IUD, which can vary in size, and hormone release, and perhaps impact the pain perceived by the patient, thus revealing an additional factor that should be considered for standardization. Disadvantages of anesthetics, such as side effects, costs, and additional pain during application, were also not included in the review. The perception of pain is inherently individualized and nuanced, rendering it a challenging parameter to accurately quantify and report. Notwithstanding the common utilization of only two scale formats in this review, namely the VAS and the Numerical Rating Scale by patients, the subjective nature of these scales introduces a level of uncertainty regarding their precision and equivalency. Additionally, there is little data evaluating the pain experienced in the days following IUD insertion. Experiencing pain immediately and multiple days after the procedure could impact the desire to choose an IUD as a form of contraception.

## Conclusions

After an exploration of the articles in this review regarding IUD pain management, there appears to be a lack of standardized protocols across practices. The absence of consistency may be due to circumstantial factors and variations in non-pharmacological and pharmacological approaches. Circumstantial factors suggest that pain is an individualized experience influenced by race, past sexual and gynecologic history, and lactation patterns. While these elements may be an obstacle in determining a universal pain management protocol, the efficacy of nonpharmacological and pharmacological methods must be considered. Testing different insertion methods and tools only showed statistically significant results for a direct method. Pharmacological methods explored various medications and applications, finding that local or direct application was more successful than systemic. There is also a need for rigorous research on factors that may exacerbate pain during IUD insertion, including psychological factors and anticipated pain. Assessing multifaceted components may provide a deeper understanding of the complexity of pain management for IUD insertion. Although IUDs have been in use before the reversal of Roe v. Wade, the legal changes in the U.S. regarding abortion have created more interest in safe and efficacious contraceptive methods. More nuanced studies are needed to close these gaps in knowledge and further evaluate a standardized method of pain management. With pain proving to be a crucial barrier, it may be important to create best practice protocols for mitigating IUD-associated pain to provide women with individualized, holistic reproductive care.
