# Correction: Differing Approaches to Pain Management for Intrauterine Device Insertion and Maintenance: A Scoping Review

**DOI:** 10.7759/cureus.c323

**Published:** 2025-09-25

**Authors:** Mayisah Rahman, Connor King, Rosie Saikaly, Maria Sosa, Kristel Sibaja, Brandon Tran, Simon Tran, Pamella Morello, Se Yeon Seo, Yi Yeon Seo, Robin J Jacobs

**Affiliations:** 1 Medicine, Dr. Kiran C. Patel College of Osteopathic Medicine, Nova Southeastern University, Fort Lauderdale, USA

This article has been corrected to fix the following error in the PRISMA diagram: "Reports excluded not relevant to IUD pain management protocols (n = 5)" has been corrected to "(n = 4)"

